# The genome sequence of a digger wasp, *Ectemnius continuus *(Fabricius, 1804)

**DOI:** 10.12688/wellcomeopenres.20138.1

**Published:** 2023-10-18

**Authors:** Liam M. Crowley, University of Oxford and Wytham Woods Genome Acquisition Lab, Darwin Tree of Life Barcoding collective, Wellcome Sanger Institute Tree of Life programme, Wellcome Sanger Institute Scientific Operations: DNA Pipelines collective, Tree of Life Core Informatics collective, Darwin Tree of Life Consortium

**Affiliations:** 1 Department of Biology, University of Oxford, Oxford, England, UK

**Keywords:** Ectemnius continuus, digger wasp, genome sequence, chromosomal, Hymenoptera

## Abstract

We present a genome assembly from an individual female *Ectemnius continuus* (digger wasp; Arthropoda; Insecta; Hymenoptera; Crabronidae). The genome sequence is 260.3 megabases in span. Most of the assembly is scaffolded into 14 chromosomal pseudomolecules. The mitochondrial genome has also been assembled and is 27.05 kilobases in length. Gene annotation of this assembly on Ensembl identified 9,835 protein coding genes.

## Species taxonomy

Eukaryota; Metazoa; Eumetazoa; Bilateria; Protostomia; Ecdysozoa; Panarthropoda; Arthropoda; Mandibulata; Pancrustacea; Hexapoda; Insecta; Dicondylia; Pterygota; Neoptera; Endopterygota; Hymenoptera; Apocrita; Aculeata; Apoidea; Crabronidae; Crabroninae; Crabronini; Crabronina; *Ectemnius*; *Ectemnius continuus* (Fabricius, 1804) (NCBI:txid1126389).

## Background

*Ectemnius continuus* is a small to medium sized digger wasp in the family Crabronidae. It is widespread throughout the Holarctic, and in the UK it is a common species, particularly across the south. It is black with yellow markings on the scapes, pronotum, tibiae and tergites. It is one of two British species of *Ectemnius* with reduced yellow marking on tergites 1 and 3, and is larger and more common than the other species with this pattern, *E. rubicola*. The clypeus is covered with shining silver hairs. Unusually for aculeates, the male *Ectemnius* do not have an additional antennal segment, with both sexes possessing 12 segments. Male *E. continuus* have unique small spines on the first and second tarsomeres of the mid tarsus.

It occurs in a wide range of habitats including woodlands, gardens and farmland. It is univoltine, with a flight period from early May to late September, however, it is likely to be bivoltine in the south of the UK. Females hunt medium-sized Diptera such as syrphids, muscids and calliphorids ( Archer, 1995). Tabanidae and Therevidae have also been recorded prey ( Lomholdt, 1975). Nests are constructed in cavities in dead wood, such as old beetle burrows in tree stumps and standing dead wood. Nest structure varies from straight to branching, and may contain up to 10 cells, with each cell provisioned with six to eight flies ( Lomholdt, 1975). Adults are strongly associated with the flowers of umbellifers, including angelica, hogweed, wild carrot, wild parsnip, fennel, cow parsley and water-dropwort, which they visit for both nectar and prey.

The complete genome sequence for this species will facilitate studies into the evolution of hunting strategies, reproductive systems and Hymenopteran taxonomy.

## Genome sequence report

**Figure 1.**
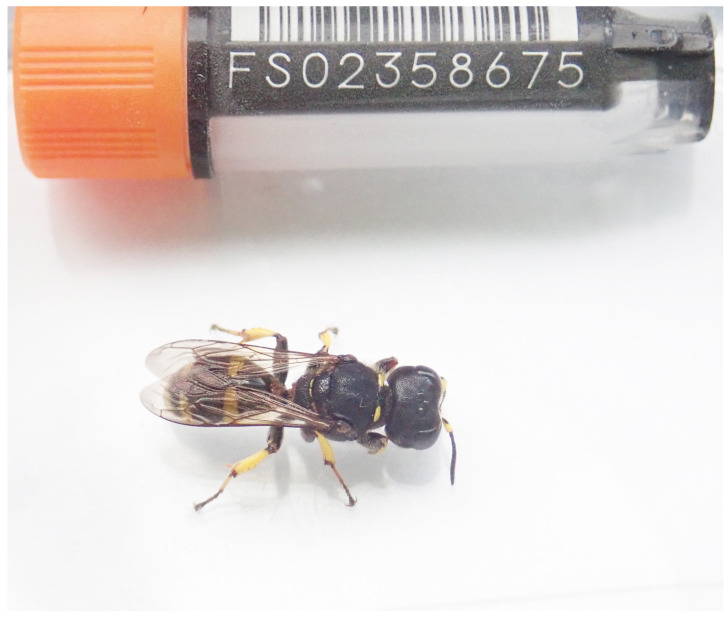
Photograph of the *Ectemnius continuus* (iyEctCont1) specimen used for genome sequencing.

The genome was sequenced from one female *Ectemnius continuus* ( Figure 1) collected from Wytham Woods, Oxfordshire, UK (51.77, –1.33). A total of 92-fold coverage in Pacific Biosciences single-molecule HiFi long reads and 132-fold coverage in 10X Genomics read clouds were generated. Primary assembly contigs were scaffolded with chromosome conformation Hi-C data. Manual assembly curation corrected 43 missing joins or mis-joins and removed 9 haplotypic duplications, reducing the assembly length by 2.65% and the scaffold number by 10.1%, and increasing the scaffold N50 by 23.41%.

**Figure 2.**
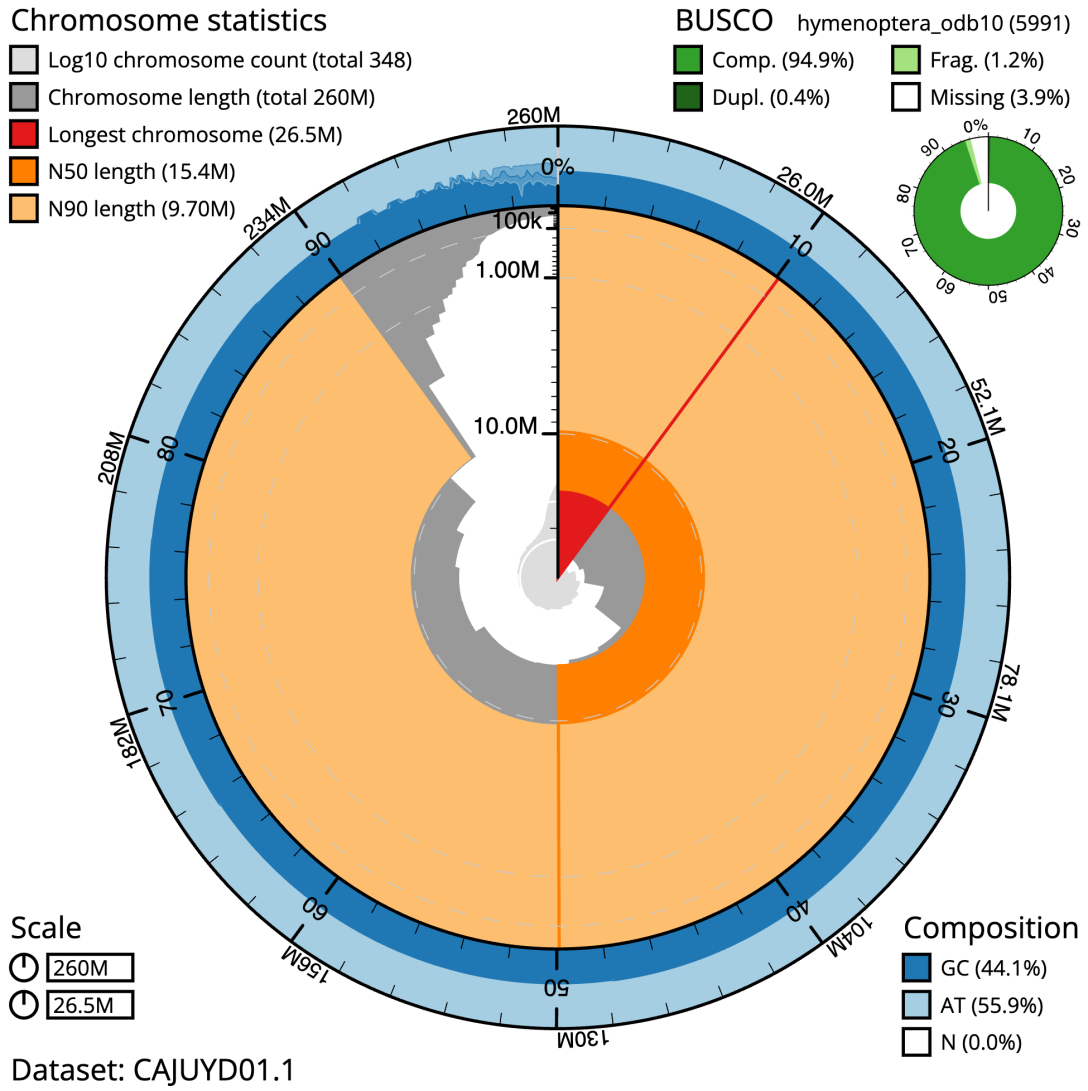
Genome assembly of *Ectemnius continuus*, iyEctCont1.1: metrics. The BlobToolKit Snailplot shows N50 metrics and BUSCO gene completeness. The main plot is divided into 1,000 size-ordered bins around the circumference with each bin representing 0.1% of the 260,364,363 bp assembly. The distribution of scaffold lengths is shown in dark grey with the plot radius scaled to the longest scaffold present in the assembly (26,542,703 bp, shown in red). Orange and pale-orange arcs show the N50 and N90 scaffold lengths (15,408,693 and 9,697,251 bp), respectively. The pale grey spiral shows the cumulative scaffold count on a log scale with white scale lines showing successive orders of magnitude. The blue and pale-blue area around the outside of the plot shows the distribution of GC, AT and N percentages in the same bins as the inner plot. A summary of complete, fragmented, duplicated and missing BUSCO genes in the hymenoptera_odb10 set is shown in the top right. An interactive version of this figure is available at https://blobtoolkit.genomehubs.org/view/iyEctCont1.1/dataset/CAJUYD01.1/snail.

**Figure 3.**
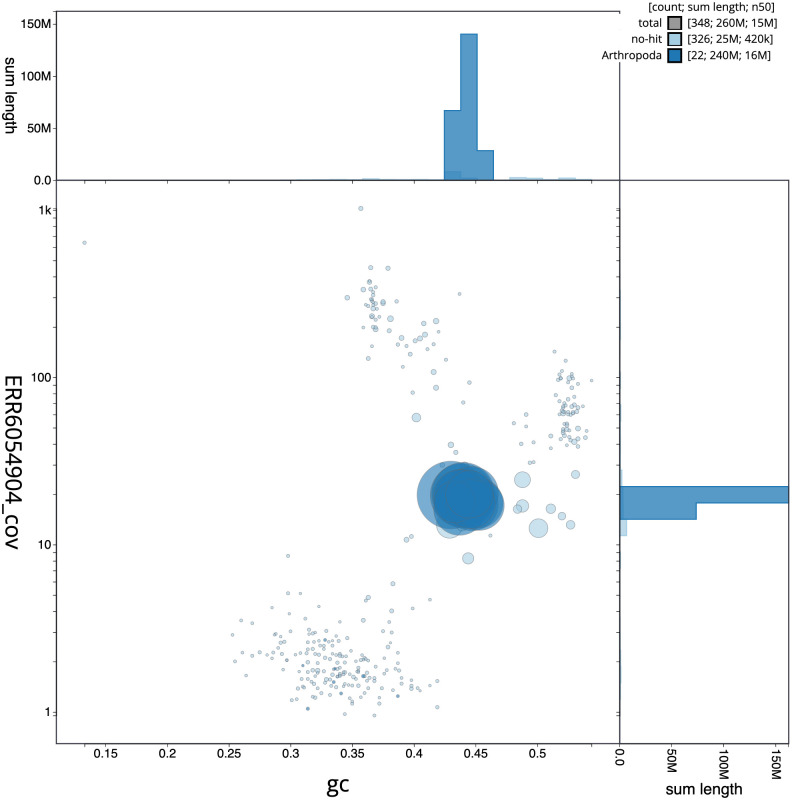
Genome assembly of *Ectemnius continuus*, iyEctCont1.1: BlobToolKit GC-coverage plot. Scaffolds are coloured by phylum. Circles are sized in proportion to scaffold length. Histograms show the distribution of scaffold length sum along each axis. An interactive version of this figure is available at https://blobtoolkit.genomehubs.org/view/iyEctCont1.1/dataset/CAJUYD01.1/blob.

**Figure 4.**
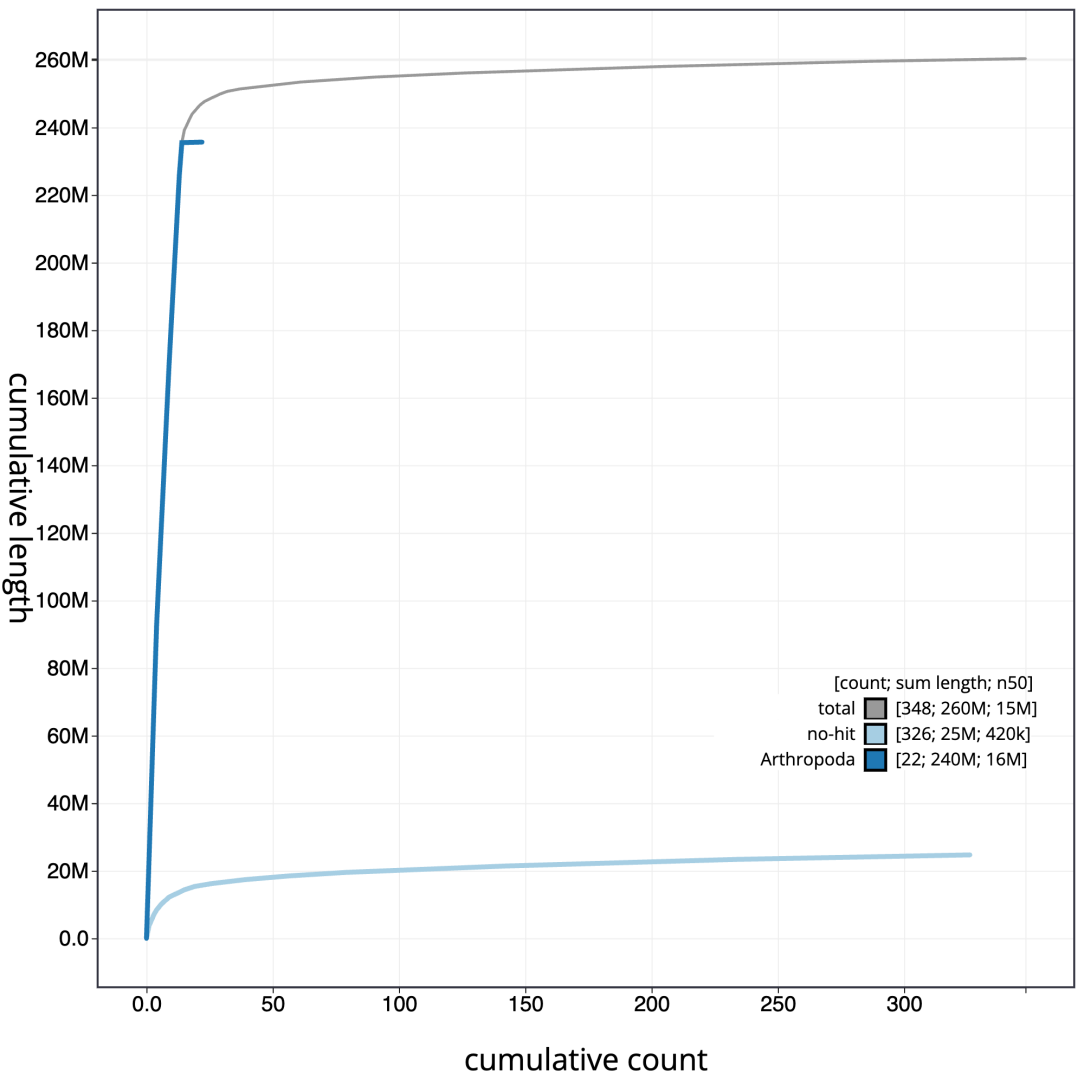
Genome assembly of *Ectemnius continuus*, iyEctCont1.1: BlobToolKit cumulative sequence plot. The grey line shows cumulative length for all scaffolds. Coloured lines show cumulative lengths of scaffolds assigned to each phylum using the buscogenes taxrule. An interactive version of this figure is available at https://blobtoolkit.genomehubs.org/view/iyEctCont1.1/dataset/CAJUYD01.1/cumulative.

**Figure 5.**
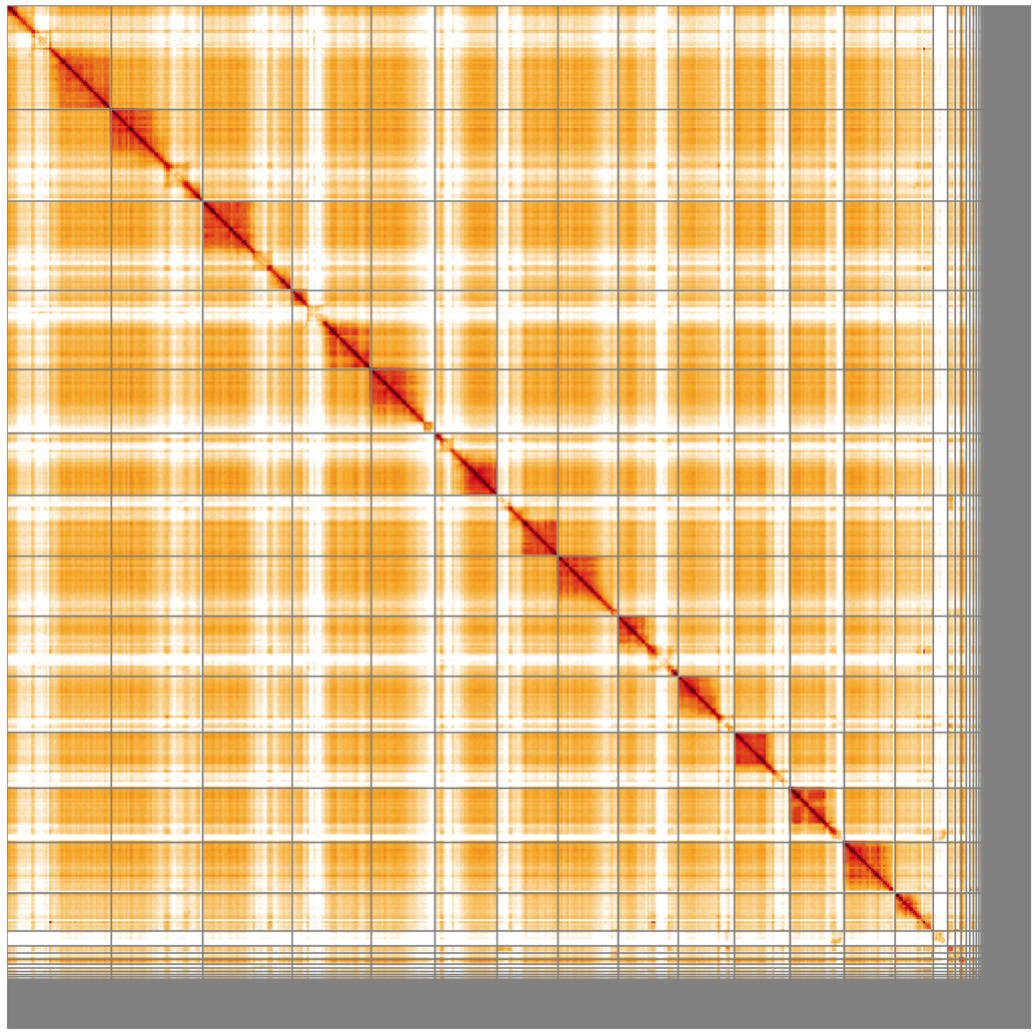
Genome assembly of *Ectemnius continuus*, iyEctCont1.1: Hi-C contact map of the iyEctCont1.1 assembly, visualised using HiGlass. Chromosomes are shown in order of size from left to right and top to bottom. An interactive version of this figure may be viewed at https://genome-note-higlass.tol.sanger.ac.uk/l/?d=ZKIZReSkS1arzsdQMBNaig.

The final assembly has a total length of 260.3 Mb in 347 sequence scaffolds with a scaffold N50 of 15.4 Mb ( Table 1). A summary of the assembly statistics is shown in Figure 2, while the distribution of assembly scaffolds on GC proportion and coverage is shown in Figure 3. The cumulative assembly plot in Figure 4 shows curves for subsets of scaffolds assigned to different phyla. Most (92.21%) of the assembly sequence was assigned to 14 chromosomal-level scaffolds. Chromosome-scale scaffolds confirmed by the Hi-C data are named in order of size ( Figure 5; Table 2). The specimen is a diploid female. While not fully phased, the assembly deposited is of one haplotype. Contigs corresponding to the second haplotype have also been deposited. The mitochondrial genome was also assembled and can be found as a contig within the multifasta file of the genome submission.

**Table 1.** Genome data for *Ectemnius continuus*, iyEctCont1.1.

Project accession data		
Assembly identifier	iyEctCont1.1	
Assembly release date	2021-07-07	
Species	*Ectemnius continuus*	
Specimen	iyEctCont1	
NCBI taxonomy ID	1126389	
BioProject	PRJEB45183	
BioSample ID	SAMEA7520490	
Isolate information	iyEctCont1, female: head and thorax (DNA sequencing); abdomen (Hi-C scaffolding)	
Assembly metrics *		*Benchmark*
Consensus quality (QV)	51.5	*≥ 50*
*k*-mer completeness	99.99%	*≥ 95%*
BUSCO **	C:94.9%[S:94.5%,D:0.4%],F:1.2%,M:3.9%,n:5,991	*C ≥ 95%*
Percentage of assembly mapped to chromosomes	92.21%	*≥ 95%*
Sex chromosomes	-	*localised homologous pairs*
Organelles	Mitochondrial genome assembled	*complete single alleles*
Raw data accessions		
PacificBiosciences SEQUEL II	ERR6560802	
10X Genomics Illumina	ERR6054902, ERR6054903, ERR6054904, ERR6054905	
Hi-C Illumina	ERR6054906, ERR6054908, ERR6054907	
Genome assembly		
Assembly accession	GCA_910591665.1	
*Accession of alternate haplotype*	GCA_910591485.1	
Span (Mb)	260.3	
Number of contigs	412	
Contig N50 length (Mb)	12.1	
Number of scaffolds	347	
Scaffold N50 length (Mb)	15.4	
Longest scaffold (Mb)	26.5	
Genome annotation		
Number of protein-coding genes	9,835	
Number of non-coding genes	1,469	
Number of gene transcripts	16,999	

* Assembly metric benchmarks are adapted from column VGP-2020 of “Table 1: Proposed standards and metrics for defining genome assembly quality” from ( Rhie *et al.*, 2021). ** BUSCO scores based on the hymenoptera_odb10 BUSCO set using v5.3.2. C = complete [S = single copy, D = duplicated], F = fragmented, M = missing, n = number of orthologues in comparison. A full set of BUSCO scores is available at https://blobtoolkit.genomehubs.org/view/iyEctCont1.1/dataset/CAJUYD01.1/busco.

**Table 2.** Chromosomal pseudomolecules in the genome assembly of *Ectemnius continuus*, iyEctCont1.

INSDC accession	Chromosome	Length (Mb)	GC%
OU342856.1	1	26.54	43.0
OU342857.1	2	23.26	44.0
OU342858.1	3	22.73	44.0
OU342859.1	4	20.12	44.0
OU342860.1	5	16.15	44.5
OU342861.1	6	15.86	44.5
OU342862.1	7	15.41	44.0
OU342863.1	8	15.33	43.5
OU342864.1	9	15.29	43.5
OU342865.1	10	14.26	45.0
OU342866.1	11	14.2	45.5
OU342867.1	12	13.76	44.5
OU342868.1	14	12.88	44.5
OU342869.1	13	9.7	43.5
OU342870.1	MT	0.03	13.5

The estimated Quality Value (QV) of the final assembly is 51.5 with *k*-mer completeness of 99.99%, and the assembly has a BUSCO v5.3.2 completeness of 94.9% (single = 94.5%, duplicated = 0.4%), using the hymenoptera_odb10 reference set ( *n* = 5,991).

Metadata for specimens, spectral estimates, sequencing runs, contaminants and pre-curation assembly statistics can be found at https://links.tol.sanger.ac.uk/species/1126389.

## Genome annotation report

The *Ectemnius continuus* genome assembly (GCA_910591665.1) was annotated using the Ensembl rapid annotation pipeline ( Table 1; https://rapid.ensembl.org/Ectemnius_continuus_GCA_910591665.1/Info/Index). The resulting annotation includes 16,999 transcribed mRNAs from 9,835 protein-coding and 1,469 non-coding genes.

## Methods

### Sample acquisition and nucleic acid extraction

A female *Ectemnius continuus* (specimen ID Ox000186, ToLID iyEctCont1) was netted in Wytham Woods, Oxfordshire (biological vice-county Berkshire), UK (latitude 51.77, longitude –1.33) on 2019-08-20. The specimen was collected and identified by Liam Crowley (University of Oxford) and preserved on dry ice.

DNA was extracted at the Tree of Life laboratory, Wellcome Sanger Institute (WSI). The iyEctCont1 sample was weighed and dissected on dry ice with tissue set aside for Hi-C sequencing. Head and thorax tissue was disrupted using a Nippi Powermasher fitted with a BioMasher pestle. High molecular weight (HMW) DNA was extracted using the Qiagen MagAttract HMW DNA extraction kit. Low molecular weight DNA was removed from a 20 ng aliquot of extracted DNA using the 0.8X AMpure XP purification kit prior to 10X Chromium sequencing; a minimum of 50 ng DNA was submitted for 10X sequencing. HMW DNA was sheared into an average fragment size of 12–20 kb in a Megaruptor 3 system with speed setting 30. Sheared DNA was purified by solid-phase reversible immobilisation using AMPure PB beads with a 1.8X ratio of beads to sample to remove the shorter fragments and concentrate the DNA sample. The concentration of the sheared and purified DNA was assessed using a Nanodrop spectrophotometer and Qubit Fluorometer and Qubit dsDNA High Sensitivity Assay kit. Fragment size distribution was evaluated by running the sample on the FemtoPulse system.

### Sequencing

Pacific Biosciences HiFi circular consensus and 10X Genomics read cloud DNA sequencing libraries were constructed according to the manufacturers’ instructions. DNA sequencing was performed by the Scientific Operations core at the WSI on Pacific Biosciences SEQUEL II (HiFi) and HiSeq X Ten (10X) instruments. Hi-C data were also generated from abdomen tissue of iyEctCont1 using the Arima2 kit and sequenced on the HiSeq X Ten instrument.

### Genome assembly, curation and evaluation

Assembly was carried out with Hifiasm ( Cheng *et al.*, 2021) and haplotypic duplication was identified and removed with purge_dups ( Guan *et al.*, 2020). One round of polishing was performed by aligning 10X Genomics read data to the assembly with Long Ranger ALIGN, calling variants with FreeBayes ( Garrison & Marth, 2012). The assembly was then scaffolded with Hi-C data ( Rao *et al.*, 2014) using SALSA2 ( Ghurye *et al.*, 2019). The assembly was checked for contamination and corrected using the gEVAL system ( Chow *et al.*, 2016) as described previously ( Howe *et al.*, 2021). Manual curation was performed using gEVAL, HiGlass ( Kerpedjiev *et al.*, 2018) and Pretext ( Harry, 2022). The mitochondrial genome was assembled using MitoHiFi ( Uliano-Silva *et al.*, 2023), which runs MitoFinder ( Allio *et al.*, 2020) or MITOS ( Bernt *et al.*, 2013) and uses these annotations to select the final mitochondrial contig and to ensure the general quality of the sequence.

A Hi-C map for the final assembly was produced using bwa-mem2 ( Vasimuddin *et al.*, 2019) in the Cooler file format ( Abdennur & Mirny, 2020). To assess the assembly metrics, the *k*-mer completeness and QV consensus quality values were calculated in Merqury ( Rhie *et al.*, 2020). This work was done using Nextflow ( Di Tommaso *et al.*, 2017) DSL2 pipelines “sanger-tol/readmapping” ( Surana *et al.*, 2023a) and “sanger-tol/genomenote” ( Surana *et al.*, 2023b). The genome was analysed within the BlobToolKit environment ( Challis *et al.*, 2020) and BUSCO scores ( Manni *et al.*, 2021; Simão *et al.*, 2015) were calculated.

Table 3 contains a list of relevant software tool versions and sources.

**Table 3.** Software tools: versions and sources.

Software tool	Version	Source
BlobToolKit	4.1.7	https://github.com/blobtoolkit/ blobtoolkit
BUSCO	5.3.2	https://gitlab.com/ezlab/busco
FreeBayes	1.3.1-17- gaa2ace8	https://github.com/freebayes/ freebayes
gEVAL	N/A	https://geval.org.uk/
Hifiasm	0.15	https://github.com/chhylp123/ hifiasm
HiGlass	1.11.6	https://github.com/higlass/ higlass
Long Ranger ALIGN	2.2.2	https://support.10xgenomics. com/genome-exome/software/ pipelines/latest/advanced/other- pipelines
Merqury	MerquryFK	https://github.com/ thegenemyers/MERQURY.FK
MitoHiFi	2	https://github.com/ marcelauliano/MitoHiFi
PretextView	0.2	https://github.com/wtsi-hpag/ PretextView
purge_dups	1.2.3	https://github.com/dfguan/ purge_dups
SALSA	2.2	https://github.com/salsa-rs/salsa
sanger-tol/ genomenote	v1.0	https://github.com/sanger-tol/ genomenote
sanger-tol/ readmapping	1.1.0	https://github.com/sanger-tol/ readmapping/tree/1.1.0

### Genome annotation

The Ensembl gene annotation system ( Aken *et al.*, 2016) was used to generate annotation for the *Ectemnius continuus* assembly (GCA_910591665.1). Annotation was created primarily through alignment of transcriptomic data to the genome, with gap filling via protein-to-genome alignments of a select set of proteins from UniProt ( UniProt Consortium, 2019).

### Wellcome Sanger Institute – Legal and Governance

The materials that have contributed to this genome note have been supplied by a Darwin Tree of Life Partner. The submission of materials by a Darwin Tree of Life Partner is subject to the **‘Darwin Tree of Life Project Sampling Code of Practice’**, which can be found in full on the Darwin Tree of Life website here. By agreeing with and signing up to the Sampling Code of Practice, the Darwin Tree of Life Partner agrees they will meet the legal and ethical requirements and standards set out within this document in respect of all samples acquired for, and supplied to, the Darwin Tree of Life Project. 

Further, the Wellcome Sanger Institute employs a process whereby due diligence is carried out proportionate to the nature of the materials themselves, and the circumstances under which they have been/are to be collected and provided for use. The purpose of this is to address and mitigate any potential legal and/or ethical implications of receipt and use of the materials as part of the research project, and to ensure that in doing so we align with best practice wherever possible. The overarching areas of consideration are:

• Ethical review of provenance and sourcing of the material

• Legality of collection, transfer and use (national and international) 

Each transfer of samples is further undertaken according to a Research Collaboration Agreement or Material Transfer Agreement entered into by the Darwin Tree of Life Partner, Genome Research Limited (operating as the Wellcome Sanger Institute), and in some circumstances other Darwin Tree of Life collaborators.

## Data Availability

European Nucleotide Archive: *Ectemnius continuus*. Accession number PRJEB45183; https://identifiers.org/ena.embl/PRJEB45183. ( Wellcome Sanger Institute, 2021) The genome sequence is released openly for reuse. The *Ectemnius continuus* genome sequencing initiative is part of the Darwin Tree of Life (DToL) project. All raw sequence data and the assembly have been deposited in INSDC databases. Raw data and assembly accession identifiers are reported in Table 1.

## Author information

Members of the University of Oxford and Wytham Woods Genome Acquisition Lab are listed here: https://doi.org/10.5281/zenodo.4789928.

Members of the Darwin Tree of Life Barcoding collective are listed here: https://doi.org/10.5281/zenodo.4893703.

Members of the Wellcome Sanger Institute Tree of Life programme are listed here: https://doi.org/10.5281/zenodo.4783585.

Members of Wellcome Sanger Institute Scientific Operations: DNA Pipelines collective are listed here: https://doi.org/10.5281/zenodo.4790455.

Members of the Tree of Life Core Informatics collective are listed here: https://doi.org/10.5281/zenodo.5013541.

Members of the Darwin Tree of Life Consortium are listed here: https://doi.org/10.5281/zenodo.4783558.
