# Supplementary material for: The genome sequence of a digger wasp, Ectemnius continuus (Fabricius, 1804)
Source: Wellcome Open Res. 2023 Oct 18;8:469. [Version 1] doi: 10.12688/wellcomeopenres.20138.1 (PMC10964002; doi:10.12688/wellcomeopenres.20138.1)
Supplement: Peer review history

# Reviewer report: Reviewer response for version 1

**DOI:** 10.21956/wellcomeopenres.22300.r76301

**Published:** 2024-03-25

**Authors:** Rodolpho S.T. Menezes

**Affiliations:** 1 State University of Santa Cruz, Ilhéus, Brazil

The manuscript by Crowley presents a genome assembly from a female of the digger wasp, *Ectemnius continuus *(Fabricius, 1804). The final assembly is 260.3 megabases and most of the assembly is scaffolded into 14 chromosomal pseudomolecules.

 Overall the manuscript presents an advance in the genomic field of wasps. This genome information will be interesting for comparative genomics investigation and also for evolution and conservation studies. The methodology is thorough and explained and the figures are well constructed and the manuscript is well written.

 However, I think the author should compare its results with the assembled genomes from other wasp species, with attention for the mitochondrial genome. The assembled mitogenome was 27.05 kilobases in length, it is unusual for wasps in general.

 Minor point: Since the author assembled the mitochondrial genome, I think it is interesting to show general (GC/AT content…) mitochondrial information about this species.

Are sufficient details of methods and materials provided to allow replication by others?

Yes

Is the rationale for creating the dataset(s) clearly described?

Yes

Are the datasets clearly presented in a useable and accessible format?

Partly

Are the protocols appropriate and is the work technically sound?

Yes

Reviewer Expertise:

Phylogenomics, Cytogenetics, and Phylogeography.

I confirm that I have read this submission and believe that I have an appropriate level of expertise to confirm that it is of an acceptable scientific standard, however I have significant reservations, as outlined above.

# Reviewer report: Reviewer response for version 1

**DOI:** 10.21956/wellcomeopenres.22300.r76299

**Published:** 2024-03-25

**Authors:** Yuanmeng Miles Zhang

**Affiliations:** 1 The University of Edinburgh, Edinburgh, Scotland, UK

The authors report the chromosome scale assembly of the genome sequence of the crabronid wasp, *Ectemnius continuus*, obtained using state-of-the-art methodologies. The background provides important information on the life history of the species including interactions with its hosts.

 Similar to other genome notes from this group, the genome sequence is of very high quality and will be a valuable source for comparative genetics, evolutionary biology, and phylogenomic studies.

Are sufficient details of methods and materials provided to allow replication by others?

Yes

Is the rationale for creating the dataset(s) clearly described?

Yes

Are the datasets clearly presented in a useable and accessible format?

Yes

Are the protocols appropriate and is the work technically sound?

Yes

Reviewer Expertise:

Phylogenomics, systematics, evolutionary ecology

I confirm that I have read this submission and believe that I have an appropriate level of expertise to confirm that it is of an acceptable scientific standard.

# Reviewer report: Reviewer response for version 1

**DOI:** 10.21956/wellcomeopenres.22300.r76307

**Published:** 2024-03-25

**Authors:** Joseph Guhlin

**Affiliations:** 1 University of Otago, Dunedin, Otago, New Zealand

This data note present the near complete genome sequence of Ectemnius continuus. The geographic spread of the species, as well as the unique phyiscal characteristics, and nests, prey, and associated flowers are well described. Construction of the genome, as well as the excellent statistics are provided. I have no notes to add.

Are sufficient details of methods and materials provided to allow replication by others?

Yes

Is the rationale for creating the dataset(s) clearly described?

Yes

Are the datasets clearly presented in a useable and accessible format?

Yes

Are the protocols appropriate and is the work technically sound?

Yes

Reviewer Expertise:

Comparative genomics, genome assembly, population genetics, multiple species (Plants, Hymenoptera, Birds)

I confirm that I have read this submission and believe that I have an appropriate level of expertise to confirm that it is of an acceptable scientific standard.
